# Type material of Acanthocephala, Nematoda and other non-helminths phyla (Cnidaria, Annelida, and Arthropoda) housed in the Helminthological Collection of the Oswaldo Cruz Institute/ FIOCRUZ (CHIOC), Rio de Janeiro, Brazil, from 1979 to 2016

**DOI:** 10.3897/zookeys.711.14753

**Published:** 2017-10-23

**Authors:** Daniela A. Lopes, Delir Corrêa Gomes, Marcelo Knoff

**Affiliations:** 1 Laboratório de Helmintos Parasitos de Vertebrados, Instituto Oswaldo Cruz, FIOCRUZ, Av. Brasil, 4365 Rio de Janeiro, RJ, Brazil

**Keywords:** Acanthocephalans, annelids, catalogue, copepods, holotype, myxozoans, nematodes, parasites, paratype

## Abstract

The third part of the catalogue of type material in the Helminthological Collection of the Oswaldo Cruz Institute/FIOCRUZ (CHIOC), comprising types deposited between 1979 and 2016, is presented to complement the first list of all types that was published in 1979. This part encompasses Acanthocephala, Nematoda and the other non-helminth phyla Cnidaria, Annelida, and Arthropoda. Platyhelminthes was covered in the first (Monogenoidea) and second (Rhabditophora Trematoda and Cestoda) parts of the catalogue published in September 2016 and March 2017, respectively. The present catalogue comprises type material for 116 species distributed across five phyla, nine classes, 50 families, and 80 genera. Specific names are listed systematically, followed by type host, infection site, type locality, and specimens with their collection numbers and references. Species classification and nomenclature are updated.

## Introduction

The century-old Helminthological Collection of the Oswaldo Cruz Institute/FIOCRUZ (CHIOC), Rio de Janeiro, Brazil, contains helminths that form part of the fauna of Brazil, and other countries, and are from a wide range of hosts captured in a variety of different biomes. The samples are holotypes, paratypes, and representative specimens of Platyhelminthes, Acanthocephala, Nematoda, and other non-helminth phyla. The CHIOC holds around 38,400 samples of helminth parasites from South America and other continents, and represents the largest such collection in Latin America and one of the largest collections worldwide (Rego 1982, Knoff et al. 2010). Details about the history and composition of CHIOC were presented in Lopes et al. (2016).

The first catalogue of all type material held in the CHIOC recorded 719 types of helminths (only holotypes or type series): 408 nematodes, 216 digenetic trematodes, 11 monogenoids, 52 acanthocephalans, 28 cestodes, and four of pentastomids (Rego et al. 1979). Since its publication, the collection has grown substantially with the number of types increasing significantly as well. Recently, Lopes et al. (2016, 2017) published another catalogue listing 203 type species of Monogenoidea and 104 type species of the other three classes of platyhelminths (Rhabditophora, Trematoda, and Cestoda). Thus, the present catalogue is the third list of type species held in this collection, and encompasses those of Acanthocephala, Nematoda, and the non-helminth phyla Cnidaria, Annelida and Arthropoda that have been deposited in CHIOC since 1979. The purpose of this article is to inform the scientific community about the types deposited in CHIOC as of December 1, 2016, and follows the articles of the International Code of Zoological Nomenclature (ICZN 1999).

## Materials and methods

The specimens are stored in glass or plastic vials in 70% ethanol (with or without 5% glycerin), 4–10% formaldehyde (with or without 2% acetic acid), 70% ethanol-formaldehyde-acetic acid (AFA) or as microscope slide preparations. All the material is available for consultation, but holotypes are not loaned. Unless otherwise stated, all type material is in good condition.

The catalogue is arranged taxonomically as phyla, classes, orders, families, genera, and species, under the original spelling and combinations. Phyla and classes are arranged phylogenetically, starting with helminth phyla. Orders, families, genera, and species are arranged alphabetically. The information on each entry is presented in the following format:

1. Original genus-species combination with author(s) and year of publication. An asterisk (*) denotes the type species of the genus.

2. Type host: updated scientific name, author(s) and year, with original scientific name in square brackets (when changed), followed by principal taxonomic group in parentheses.

3. Infection site in the host.

4. Type locality: country, province or state, department, specific locality and coordinates (if available).

5. Primary type status: sex (if applicable/ possible), CHIOC catalog number. Categories for types follow articles 73–75 of the Code (ICZN 1999).

6. Remark sections are inserted when necessary and include additional information about host, locality, or status of the types.

7. References include publications in which the species was described and those that mention type specimens in the CHIOC.

The valid names adopted for parasitized hosts follow specific bibliographies. Diplopod names are in accordance with Pérez-Asso (1996); beetles with Boucher (2005); crustaceans with Young (1998); fishes with Froese and Pauly (2016); amphibians with Frost (2016); reptiles with Uetz et al. (2016); birds with Lepage and Warnier (2014); and mammals with Wilson and Reeder (2005). Mention of acanthocephalans, nematodes, myxozoans, annelids, copepods, and host species in this list does not imply that the authors of the present report agree with their validity or taxonomy. Some species catalogued have been synonymized and the comments about their taxonomy are provided in the remark sections. The higher classification of Acanthocephala follows Amin (1985, 2013); Nematoda follows De Ley and Blaxter (2002, 2004); Myxozoa follows Fiala et al. (2015); Annelida follows Beesley et al. (2000) and Sawyer (1986); and Copepoda follows Martin and Davis (2001). The classification of families and genera follows specific references.

### Abbreviation list

**BMNH** British Museum of Natural History, Collection at the Department of Zoology, Natural History Museum, London, England;

**CHFC** Helminthological Collection of the Science College, University of the Republic, Montevideo, Uruguay;

**CHIBB** Reference Helminthological Collection of the Parasitology Department, Bioscience Institute, Paulista State University (UNESP), Botucatu, São Paulo, Brazil;

**CHIOC** Helminthological Collection of the Oswaldo Cruz Institute, FIOCRUZ, Rio de Janeiro, Brazil;

**CHIP–URG** Helminthological Collection of the Laboratory of Ichthyoparasitology, University of Rio Grande, Rio Grande, Rio Grande do Sul, Brazil;

**CHMLP/MLP** Helminthological Collection of the Museum of La Plata, La Plata, Buenos Aires, Argentina;

**CMNPA** Canadian Museum of Nature Invertebrate Collections-Parasites, Ottawa, Canada;

**CNHE** National Collection of Helminths, Institute of Biology, National Autonomous University of Mexico, Mexico City, Mexico;

**CZACC** Helminthological Collection of the Zoological Collections, Institute of Ecology and Systematic, Havana, Cuba;

**FCAV/UNESP** Helminthological Collection of the Parasitic Diseases Laboratory, Department of Preventive Veterinary Medicine, Paulista State University, São Paulo, Brazil;

**HWML** Harold W. Manter Laboratory, University of Nebraska State Museum, Lincoln, Nebraska, USA;

**INPA** National Institute for Amazon Research, Manaus, Amazonas, Brazil;

**IPCAS/IPCR/ASCR** Institute of Parasitology, Academy of Sciences of the Czech Republic, České Budějovice, Czech Republic;

**MNHN/INVE** National Museum of Natural History, Paris, France;

**MNRJ** Carcinological Collection of the National Museum of Rio de Janeiro, Rio de Janeiro, Brazil;

**RIT** Royal Belgian Institute of Natural Sciences, Brussels, Belgium;

**RMCA** Collection of the Royal Museum of Central Africa, Tervuren, Belgium;

**USNM** United States National Museum, Smithsonian Institution, Washington, D.C, USA;

**USNPC** United States National Parasitological Collection, Beltsville, Maryland, USA.

## Results and discussion

This database and bibliographic survey provides the diversity types of Acanthocephala, Nematoda and other non-helminth phyla, such as Cnidaria (Myxozoa), Annelida (Polychaeta and Hirudinea) and Arthropoda (Copepoda), in CHIOC, from Brazil and other countries of the world, for a period of more than 35 years of parasitological studies. This catalogue adds 38 primary types of Acanthocephala, represented by eight species distributed among eight families and eight genera; approximately 420 primary types of Nematoda, represented by 99 species, distributed across 36 families and 64 genera; four primary types of Myxozoa, represented by one family, two genera and two species; eight primary types of one species of Polychaeta and six primary types of one species of Hirudinea; and 22 primary types of Copepoda, represented by five species, distributed in three families, and four genera. The most representative families were the nematode families Cucullanidae, with 18 species, followed by Pharyngodonidae, with seven.

One hundred and sixteen parasites of 101 species of vertebrate and eight species of invertebrate hosts were catalogued. The invertebrate hosts were beetles, diplopods, and crustaceans. Most of the type species recorded (47%) were parasites of bony fishes, such as those of Monogenoidea, Trematoda, and Cestoda (Lopes et al. 2016, 2017). Only one species was a parasite of cartilaginous fishes. The hosts of parasite species of Tetrapoda were frogs, birds, reptiles, and mammals. Cricetidae (Rodentia) was the host family that exhibited the greatest diversity of parasites, with ten species.

Among the Acanthocephala listed, five species were parasites of bony fishes, one species was a parasite of frogs (Bufonidae), one species was a parasite of birds (Phalacrocoracidae), and one species was a parasite of crab-eating foxes (Canidae). Among the nematodes, five species were parasites of beetles (Passalidae) and two were parasites of diplopods (Rhinocricidae and Spirobolellidae). Species of nematode parasites of fishes included 41 of bony fishes and only one of cartilaginous fishes (Arhynchobatidae). Three nematode species were parasites of frogs (Bufonidae). The nematodes parasites of reptiles included five species parasitic on the suborder Autarchoglossa (Gymnophthalmidae, Scincidae and Teiidae), five parasites of snakes (Boidae, Colubridae, and Viperidae), and three parasites of lizards (Hoplocercidae, Liolaemidae, and Tropiduridae). Nine nematode species were parasites of birds (Ardeidae, Bucconidae, Cuculidae, Dendrocolaptidae, Opisthocomidae, Threskiornithidae, Tinamidae, and Tyrannidae). Species of nematode parasites of mammals included 16 parasites of rodents (Caviidae, Cricetidae, Dasyproctidae, Echimyidae, and Muridae), four parasites of the order Carnivora (Canidae, Felidae, and Mustelidae), two parasites of armadillos (Dasypodidae), two parasites of opossums (Didelphidae), and one parasite of bats (Molossidae). Myxozoans and copepods parasitized bony fishes. The single polychaeta species was a parasite of freshwater crayfish (Parastacidae) and the single Hirudinea species was a parasite of turtles (Podocnemididae).

Most of the species catalogued were collected in Brazil (88%), coming from all regions of the country. Other countries with cataloged material are from America (United States, Mexico, Panama, Colombia, Venezuela, Bolivia, and Argentina), the Caribbean (Cuba) and Africa (Democratic Republic of Congo). This third part of the catalogue of type material housed in CHIOC from 1979 to 2016 includes, in total, 423 species of parasites from 251 different hosts caught in almost all Brazilian states, and almost all continents, with the exception of the Middle East and Oceania (Lopes et al. 2016, 2017). Most of the deposits were made by Brazilian researchers, but foreigners also made a significant contribution, expanding and diversifying the country’s known parasitological heritage. All these data testify to the importance of CHIOC and its recognition on the world stage as a depositary, and serve as testimony of helminth parasite biodiversity, thereby placing CHIOC among the most significant collections of the world.

### List of type species

#### Phylum Acanthocephala Kohlreuther, 1771

##### Class Archiacanthocephala Meyer, 1931

###### Order Oligacanthorhynchida Petrochenko, 1956

####### Oligacanthorhynchidae Southwell & Macfie, 1925

######## *Prosthenorchis* Travassos, 1915

######### Prosthenorchis cerdocyonis

Taxon classificationAnimaliaOligacanthorhynchidaOligacanthorhynchidae

Gomes, Olifiers, Souza, Barbosa, D’Andrea, & Maldonado Jr., 2015

########## Type host.

*Cerdocyon thous* (Linnaeus, 1766) (Carnivora: Canidae).

########## Infection site.

Small intestine.

########## Type locality.

Brazil, Mato Grosso do Sul State, Nhumirim Ranch (18°59'S, 56°39'W).

########## Holotype.

♂ CHIOC 35804 a.

########## Paratypes.

CHIOC 35804 b (allotype ♀), 30812 c (5♀♀, 6♂♂).

########## Reference.

Gomes et al. (2015).

##### Class Eoacanthocephala Van Cleave, 1936

###### Order Gyracanthocephala Van Cleave, 1936

####### Quadrigyridae Van Cleave, 1920

######## *Machadosentis* Noronha, 1992

######### * Machadosentis travassosi

Taxon classificationAnimaliaGyracanthocephalaQuadrigyridae

Noronha, 1992

########## Type host.

*Gymnothorax ocellatus* Agassiz, 1831 (Osteichthyes: Muraenidae).

########## Infection site.

Intestine.

########## Type locality.

Brazil, Bahia State, Arembepe.

########## Syntypes.

CHIOC 32742 a–b (♂♂), c (♀).

########## Remarks.

CHIOC 32742 a–c referred as type material in the original description.

########## Reference.

Noronha (1992).

###### Order Neoechinorhynchida Southwell & MacFie, 1925

####### Neoechinorhynchidae (Ward, 1917) Van Cleave, 1928

######## *Neochinorhynchus* Stiles & Hassal, 1905

######### Neoechinorhynchus pimelodi

Taxon classificationAnimaliaNeoechinorhynchidaNeoechinorhynchidae

Brasil-Sato & Pavanelli, 1998

########## Type host.

*Pimelodus maculatus* Lacépède, 1803 (Osteichthyes: Pimelodidae).

########## Infection site.

Anterior intestine.

########## Type locality.

Brazil, Minas Gerais State, São Francisco River basin, Três Marias.

########## Holotype.

♂ CHIOC 33718 a.

########## Paratypes.

CHIOC 33718 b (allotype ♀), c, e (♂♂), d (♀).

########## Remarks.

Other paratypes deposited in USNPC.

########## Reference.

Brasil-Sato and Pavanelli (1998).

##### Class Palaeacanthocephala Meyer, 1931

###### Order Echinorhynchida Southwell & MacFie, 1925

####### Arhythmacanthidae Yamaguti, 1935

######## *Heterosentis* Van Cleave, 1931

######### Heterosentis brasiliensis

Taxon classificationAnimaliaEchinorhynchidaArhythmacanthidae

Vieira, Felizardo & Luque, 2009

########## Type host.

*Pseudopercis numida* Miranda-Ribeiro, 1903 (Osteichthyes: Pinguipedidae).

########## Infection site.

Intestine.

########## Type locality.

Brazil, Rio de Janeiro State, Cabo Frio (22°52'43.26"S, 42°1'11.55"W).

########## Holotype.

♂ CHIOC 37103.

########## Paratypes.

CHIOC 36672 (♂), 37104 (allotype ♀), 37105 (♀).

########## Reference.

Vieira et al. (2009).

####### Diplosentidae Tubangui & Masilungan, 1937

######## *Amapacanthus* Salgado-Maldonado & Santos, 2000

######### * Amapacanthus amazonicus

Taxon classificationAnimaliaEchinorhynchidaDiplosentidae

Salgado-Maldonado & Santos, 2000

########## Type host.

*Sciades passany* (Valenciennes, 1840) [= *Arius passany*] (Osteichthyes: Ariidae).

########## Infection site.

Intestine.

########## Type locality.

Brazil, Amapá State, Maraca Island (2°0'N, 50°24'W).

########## Holotype.

♂ CHIOC 34199 a.

########## Paratypes.

CHIOC 34199 b (allotype ♀), c–j (♀♀).

########## Remarks.

Other paratypes deposited in CNHE and BMNH.

########## Reference.

Salgado-Maldonado and Santos (2000).

####### Echinorhynchidae Cobbold, 1876

######## *Acanthocephalus* Koelreuter, 1771

######### Acanthocephalus ula

Taxon classificationAnimaliaEchinorhynchidaEchinorhynchidae

Lent & Santos, 1989

########## Type host.

*Atelopus oxyrhynchus* Boulenger, 1903 (Amphibia: Bufonidae).

########## Infection site.

Small intestine.

########## Type locality.

Venezuela, Mérida.

########## Holotype.

♂ CHIOC 32173.

########## Paratypes.

CHIOC 31667, 32174 (allotype ♀), 32175 a–b (♂♂).

########## Reference.

Lent and Santos (1989).

####### Gymnorhadinorhynchidae Braicovich, Lanfranchi, Farber, Marvaldi, Luque & Timi, 2014

######## *Gymnorhadinorhynchus* Braicovich, Lanfranchi, Farber, Marvaldi, Luque & Timi, 2014

######### * Gymnorhadinorhynchus decapteri

Taxon classificationAnimaliaEchinorhynchidaGymnorhadinorhynchidae

Braicovich, Lanfranchi, Farber, Marvaldi, Luque & Timi, 2014

########## Type host.

*Decapterus punctatus* (Cuvier, 1829) (Osteichthyes: Carangidae).

########## Infection site.

Intestine.

########## Type locality.

Brazil, Rio de Janeiro State, Cabo Frio (22°53'S, 42°00'W).

########## Holotype.

♂ CHIOC 35941 a.

########## Paratypes.

CHIOC 35941 b (allotype ♀), 35941 c (♂), d–e (♀♀).

########## Remarks.

Two additional paratypes deposited in the IPCAS collection.

########## Reference.

Braicovich et al. (2014).

###### Order Polymorphida Petrochenko, 1956

####### Polymorphidae Meyer, 1931

######## *Andracantha* Schmidt, 1975

######### Andracantha tandemtesticulata

Taxon classificationAnimaliaPolymorphidaPolymorphidae

Monteiro, Amato & Amato, 2006

########## Type host.

*Phalacrocorax brasilianus* (Gmelin, 1789) (Aves: Phalacrocoracidae).

########## Infection site.

Small and large intestine.

########## Type locality.

Brazil, Rio Grande do Sul State, Guaíba Lake, Guaíba (30°00'S, 51°15'W).

########## Holotype.

♂ CHIOC 36630 a.

########## Paratypes.

CHIOC 36630 b (allotype ♀), 36630 c (♀).

########## Reference.

Monteiro et al. (2006).

#### Phylum Nematoda Potts, 1932

##### Class Enoplea Inglis, 1983

###### Order Trichinellida Hall, 1916

####### Capillariidae Railliet, 1915

######## *Capillostrongyloides* Freitas & Lent, 1935

######### Capillostrongyloides arapaimae

Taxon classificationAnimaliaTrichinellidaCapillariidae

Santos, Moravec & Venturieri, 2008

########## Type host.

*Arapaima gigas* (Schinz, 1822) (Osteichthyes: Arapaimidae).

########## Infection site.

Anterior part of intestine and pyloric caeca.

########## Type locality.

Brazil, Pará State, Mexiana Island (Amazon River delta), natural canals and breeding tanks of fish farm at Santo Ambrósio Farm (00°05'30"S, 49°34'50"W).

########## Holotype.

♂ CHIOC 35559 a.

########## Paratypes.

CHIOC 35559 b (allotype ♀), 35559 c–d (♂, ♀).

########## Reference.

Santos et al. (2008a).

######## Pseudocapillaria (Ichthyocapillaria) Moravec, 1982

######### Pseudocapillaria (Ichthyocapillaria) maricaensis

Taxon classificationAnimaliaTrichinellidaCapillariidae

Rodrigues, 1992

########## Type host.

*Liolaemus lutzae* Mertens, 1938 (Iguania: Liolaemidae).

########## Infection site.

Small intestine.

########## Type locality.

Brazil, Rio de Janeiro State, Maricá.

########## Holotype.

♂ CHIOC 32796 a.

########## Paratypes.

CHIOC 32796 b (2♂♂), c (3♂♂), d (4♀♀), e (5♀♀), f (6♀♀), g (7♀♀), h (8♀♀), i (9♀♀).

########## Reference.

Rodrigues (1992).

####### Trichuridae Ransom, 1911

######## *Trichuris* Roederer, 1761

######### Trichuris thrichomysi

Taxon classificationAnimaliaTrichinellidaTrichuridae

Lopes Torres, Nascimento, Menezes, Garcia, Santos, Maldonado Jr., Miranda, Lanfredi & Souza, 2011

########## Type host.

*Thrichomys apereoides* Lund, 1839 (Rodentia: Echimyidae).

########## Infection site.

Cecum.

########## Type locality.

Brazil, Minas Gerais State, Capitão Andrade (19°02'14"S, 41°49'59"W).

########## Holotype.

♂ CHIOC 35210 a.

########## Paratypes.

CHIOC 35710 b (allotype ♀), 35710 c (♂), 37365 a–b (♂♂), 37365 c (♀).

########## Reference.

Lopes Torres et al. (2011).

######### Trichuris travassosi

Taxon classificationAnimaliaTrichinellidaTrichuridae

Gomes, Lanfredi, Pinto & Souza, 1992

########## Type host.

*Oligoryzomys nigripes* (Olfers, 1818) [= *Oryzomys nigripes*] (Rodentia: Cricetidae).

########## Infection site.

Large intestine.

########## Type locality.

Brazil, Rio Grande do Sul State, Arvorezinha (28°45'S, 52°15'W).

########## Holotype.

♂ CHIOC 32790 a.

########## Paratypes.

CHIOC 32790 b (♀), 32791 a–b, f–g, m (♀♀), c–e, h–l (♂♂).

########## Reference.

Gomes et al. (1992).

##### Class Chromadorea Inglis, 1983

###### Order Rhabditida Chitwood, 1933

####### Acuariidae Railliet, Henry & Sissoff, 1912

######## *Deliria* Vicente, Pinto & Noronha, 1980

######### * Deliria gomesae

Taxon classificationAnimaliaRhabditidaAcuariidae

Vicente, Pinto & Noronha, 1980

########## Type host.

*Pitangus sulphuratus* (Linnaeus, 1766) (Aves: Tyrannidae).

########## Infection site.

Stomach.

########## Type locality.

Brazil, Rio de Janeiro State, Rio de Janeiro, Raimundo Island.

########## Holotype.

♂ CHIOC 31780 a.

########## Paratypes.

CHIOC 31780 b–c, g–h (♀♀), d–f (♂♂).

########## Remarks.

There is no paratype “i” as indicated in the original description, which was a mistake.

########## Reference.

Vicente et al. (1980).

####### Anisakidae Railliet & Henry, 1912

######## *Goezia* Zeder, 1800

######### Goezia brasiliensis

Taxon classificationAnimaliaRhabditidaAnisakidae

Moravec, Kohn & Fernandes, 1994

########## Type host.

*Brycon hilarii* (Valenciennes, 1850) (Osteichthyes: Bryconidae).

########## Infection site.

Stomach.

########## Type locality.

Brazil, Paraná State, Paraná River, Foz do Iguaçú.

########## Paratype.

♀ CHIOC 32961.

########## Remarks.

Holotype and allotype deposited in the IPCAS collection.

########## Reference.

Moravec et al. (1994).

######### Goezia leporini

Taxon classificationAnimaliaRhabditidaAnisakidae

Martins & Yoshitoshi, 2003

########## Type host.

*Leporinus macrocephalus* Garavello & Britski, 1988 (Osteichthyes: Anostomidae).

########## Infection site.

Stomach.

########## Type locality.

Brazil, São Paulo State, Batatais.

########## Holotype.

♂ CHIOC 34675 a.

########## Paratypes.

CHIOC 34675 b (allotype ♀), 34675 c (♂♂), 34675 d (♀♀).

########## Reference.

Martins and Yoshitoshi (2003).

######## *Pseudanisakis* Layman & Borovkova, 1926

######### Pseudanisakis sulamericana

Taxon classificationAnimaliaRhabditidaAnisakidae

Santos, Lent & Gibson, 2004

########## Type host.

*Rioraja agassizii* (Müller & Henle, 1841) (Chondrichthyes: Arhynchobatidae).

########## Infection site.

Intestine.

########## Type locality.

Brazil, São Paulo State, off Ubatuba (23°30'S, 45°00'W).

########## Holotype

♂ **and allotype** ♀. CHIOC 35235.

########## Paratypes.

CHIOC 35236, 35237.

########## Remarks.

CHIOC 35237 collected from *Psammobatis extenta* (Garman, 1913) (Arhynchobatidae). Other paratypes deposited in the BMHN collection.

########## Reference.

Santos et al. (2004).

######## Raphidascaris (Sprentascaris) (Petter & Cassone, 1984) Moravec, Kohn & Fernandes, 1990

######### Raphidascaris (Sprentascaris) lanfrediae

Taxon classificationAnimaliaRhabditidaAnisakidae

Melo, Santos, Giese, Santos & Santos, 2011

########## Type host.

*Satanoperca jurupari* (Heckel, 1840) (Osteichthyes: Cichlidae).

########## Infection site.

Intestine.

########## Type locality.

Brazil, Pará State, Belém, Guamá River (01°27'21"S, 48°30'14"W).

########## Holotype

♂. CHIOC 35714 a.

########## Paratypes.

CHIOC 35714 b–c (5♀♀, 4♂♂).

########## Remarks.

Paratype from CHIOC cited as “35716” in the original description due to a mistake.

########## Reference.

Melo et al. (2011).

######## *Raphidascaroides* Railliet & Henry, 1915

######### Raphidascaroides moraveci

Taxon classificationAnimaliaRhabditidaAnisakidae

Pereira, Tavares, Scholz & Luque, 2015

########## Type host.

*Platydoras armatulus* (Valenciennes, 1840) (Osteichthyes: Doradidae).

########## Infection site.

Intestine.

########## Type locality.

Brazil, Mato Grosso do Sul State, Miranda River (19°34'S, 57°00'W).

########## Holotype

♂ **and allotype** ♀. CHIOC 36728 a.

########## Paratypes.

CHIOC 36728 b (3♂♂, 3♀♀), c (hologenophore).

########## Remarks.

Other paratypes deposited in the IPCAS collection.

########## Reference.

Pereira et al. (2015).

####### Aproctidae Yorke & Maplestone, 1926

######## *Aprocta* Linstow, 1883

######### Aprocta brevipenis

Taxon classificationAnimaliaRhabditidaAproctidae

Rodrigues & Rodrigues, 1980

########## Type host.

*Guira guira* (Gmelin, 1788) (Aves: Cuculidae).

########## Infection site.

Ocular cavity.

########## Type locality.

Brazil, São Paulo State, Ilha Seca.

########## Holotype.

♂ CHIOC 31808 a.

########## Paratypes.

CHIOC 31808 b (allotype ♀), 31808 c–d (♀♀).

########## Reference.

Rodrigues and Rodrigues (1980).

####### Ascaridiidae Travassos, 1919

######## *Ophidascaris* Baylis, 1920

######### Ophidascaris durissus

Taxon classificationAnimaliaRhabditidaAscaridiidae

Panizzutti, Santos, Vicente, Muniz-Pereira & Pinto, 2003

########## Type host.

*Crotalus durissus* Linnaeus, 1758 (Serpentes: Viperidae).

########## Infection site.

Stomach.

########## Type locality.

Brazil, Paraná State, Itaipú Binacional Reserve, Foz do Iguaçú (24°05'–25°33'S, 54°00'–54°37'W).

########## Holotype.

CHIOC 34937 a.

########## Paratypes.

CHIOC 34937 b–f.

########## Reference.

Panizzuti et al. (2003).

######### Ophidascaris tuberculatum

Taxon classificationAnimaliaRhabditidaAscaridiidae

Siqueira, Panizzutti, Muniz-Pereira & Pinto, 2005

########## Type host.

*Bothrops jararaca* (Wied-Neuwied, 1824) (Serpentes: Viperidae).

########## Infection site.

Stomach.

########## Type locality.

Brazil, Rio de Janeiro State, Petrópolis, Serra das Araras (22°30'39"S, 43°11'4"W), 857 m high.

########## Holotype.

♂ CHIOC 36232 a.

########## Paratypes.

CHIOC 35315, 36232 b (allotype ♀), 36232 c (♀), d (anterior extremity).

########## Reference.

Siqueira et al. (2005).

####### Aspidoderidae Skrjabin & Schikhobalova, 1947

######## *Aspidodera* Railliet & Henry, 1912

######### Aspidodera sogandaresi

Taxon classificationAnimaliaRhabditidaAspidoderidae

Jiménez-Ruiz, Gardner & Varela-Stokes, 2006

########## Type host.

*Dasypus novemcinctus* Linnaeus, 1758 (Cingulata: Dasypodidae).

########## Infection site.

Large intestine.

########## Type locality.

United States, Texas, El Pedregal, 18 miles north by road (U.S. 281) from Lampasas (31°19'34"N, 98°09'33"W), 311 m high.

########## Paratypes.

CHIOC 35429 (4♂♂, 4♀♀), 35430 (10♂, 10♀), 35431 (3♂♂, ♀).

########## Remarks.

There are two paratypes in CHIOC collected from Mexico, but there are some inconsistencies with regard to the collecting localities in the original description and the cataloging data. Holotype, allotype, and other paratypes are deposited in the HWML collection. Additional paratypes are deposited in CMNPA, CNHE, and USNM.

########## Reference.

Jiménez-Ruiz et al. (2006).

######### Aspidodera vicentei

Taxon classificationAnimaliaRhabditidaAspidoderidae

Pinto, Kohn, Fernandes & Mello, 1982

########## Type host.

*Nectomys squamipes* (Brants, 1827) (Rodentia: Cricetidae).

########## Infection site.

Small intestine.

########## Type locality.

Brazil, Goiás State, Formosa.

########## Holotype.

♂ CHIOC 31879 a.

########## Paratypes.

CHIOC 31879 b–f.

########## Reference.

Pinto et al. (1982).

####### Camallanidae Railliet & Henry, 1915

######## *Camallanus* Railliet & Henry, 1915

######### Camallanus acaudatus

Taxon classificationAnimaliaRhabditidaCamallanidae

Ferraz & Thatcher, 1990

########## Type host.

*Osteoglossum bicirrhosum* (Cuvier, 1829) (Osteichthyes: Osteoglossidae).

########## Infection site.

Intestine.

########## Type locality.

Brazil, Amazonas State, Anavilhanas Archipelago, Negro River.

########## Paratypes.

CHIOC 32557 a–b (♂, ♀).

########## Remarks.

Holotype and allotype deposited in the INPA collection. CHIOC numbers were not included in the original description.

########## Reference.

Ferraz and Thatcher (1990).

######### Camallanus maculatus

Taxon classificationAnimaliaRhabditidaCamallanidae

Martins, Garcia, Piazza & Ghiraldelli, 2007

########## Type host.

*Xiphophorus maculatus* (Günther, 1866) (Osteichthyes: Poeciliidae).

########## Infection site.

Intestine.

########## Type locality.

Brazil, São Paulo State, Araraquara.

########## Holotype

♂, **allotype** ♀ **and paratypes.** CHIOC 35283.

########## Reference.

Martins et al. (2007).

######## *Paracamallanus* Yorke & Maplestone, 1926

######### Paracamallanus amazonensis

Taxon classificationAnimaliaRhabditidaCamallanidae

Ferraz & Thatcher, 1992

########## Type host.

*Hypophthalmus edentatus* Spix & Agassiz, 1829 (Osteichthyes: Pimelodidae).

########## Infection site.

Intestine.

########## Type locality.

Brazil, Amazonas State, Marchantaria Island, Solimões River.

########## Paratypes.

CHIOC 32756 (♂), 32757 (♀).

########## Remarks.

Holotype, allotype and other paratypes deposited in the collection of INPA. CHIOC numbers were not included in the original description.

########## Reference.

Ferraz and Thatcher (1992).

######## Procamallanus (Procamallanus) (Baylis, 1923) Ali, 1957

######### Procamallanus (Procamallanus) annipetterae

Taxon classificationAnimaliaRhabditidaCamallanidae

Kohn & Fernandes, 1988

########## Type host.

*Hypostomus albopunctatus* (Regan, 1908) (Osteichthyes: Loricariidae).

########## Infection site.

Intestine.

########## Type locality.

Brazil, Paraná State, Iguaçú River, Hydroelectric Power station Salto Osório.

########## Holotype.

♂ CHIOC 32430 a.

########## Paratype.

CHIOC 32430 b (allotype ♀).

########## References.

Kohn and Fernandes (1988a, b).

######## Procamallanus (Spirocamallanus) (Olsen, 1952) Moravec & Sey, 1988

######### Procamallanus (Spirocamallanus) belenensis

Taxon classificationAnimaliaRhabditidaCamallanidae

Giese, Santos & Lanfredi, 2009

########## Type host.

*Ageneiosus ucayalensis* Castelnau, 1855 (Osteichthyes: Auchenipteridae).

########## Infection site.

Intestine.

########## Type locality.

Brazil, Pará State, Belém, Guajará Bay (1°15'–1°29'S, 48°32'–48°29'W).

########## Holotype.

♂ CHIOC 35604 a.

########## Paratypes.

CHIOC 35604 b (allotype ♀), 35604 c.

########## Reference.

Giese et al. (2009).

######### Procamallanus (Spirocamallanus) pintoi

Taxon classificationAnimaliaRhabditidaCamallanidae

Kohn & Fernandes, 1988

########## Type host.

*Corydoras paleatus* (Jenyns, 1842) (Osteichthyes: Callichthyidae).

########## Infection site.

Intestine.

########## Type locality.

Brazil, Paraná State, Iguaçú River, Hydroelectric Power station Salto Osório.

########## Holotype.

♂ CHIOC 32432 a.

########## Paratypes.

CHIOC 32431 a (allotype ♀), 32431 b (♂), 32432 b–c (♀♀).

########## Reference.

Kohn and Fernandes (1988a).

####### Carnoyidae Filipjev, 1934

######## *Carnoya* Gilson, 1898

######### Carnoya isabelica

Taxon classificationAnimaliaRhabditidaCarnoyidae

García & Morffe, 2014

########## Type host.

*Nesobolus piedra* Pérez-Asso, 1996 (Diplopoda: Rhinocricidae).

########## Infection site.

Hind gut.

########## Type locality.

Cuba, Santiago de Cuba province, La Gran Piedra, La Isabelica, (20°00'32.68"N, 75°37'18.8"W).

########## Paratypes.

CHIOC 38210 a–b (2♂♂, 3♀♀).

########## Remarks.

Holotype female and other paratypes males and females deposited in CZACC.

########## Reference.

García and Morffe (2014).

####### Cosmocercidae Railliet, 1916

######## *Cosmocercoides* Wilkie, 1930

######### Cosmocercoides sauria

Taxon classificationAnimaliaRhabditidaCosmocercidae

Ávila, Strüssmann & Silva, 2010

########## Type host.

*Iphisa elegans* Gray, 1851 (Autarchoglossa: Gymnophthalmidae).

########## Infection site.

Large intestine.

########## Type locality.

Brazil, Mato Grosso State, São Domingos Valley (15°07'S, 58°58'W).

########## Holotype.

♂ CHIOC 35654 a.

########## Paratype.

CHIOC 35654 b (allotype ♀).

########## Remarks.

Three paratypes deposited in CHIBB.

########## Reference.

Ávila et al. (2010).

####### Crenosomatidae Skrjabin, 1933

######## *Crenosoma* Molin, 1861

######### Crenosoma brasiliense

Taxon classificationAnimaliaRhabditidaCrenosomatidae

Vieira, Muniz-Pereira, Lima, Moraes Neto, Guimarães & Luque, 2012

########## Type host.

*Galictis cuja* (Molina, 1782) (Carnivora: Mustelidae).

########## Infection site.

Bronchi and bronchioles.

########## Type locality.

Brazil, Minas Gerais State, Juiz de Fora (21°76'S, 43°21'W).

########## Holotype.

♂ CHIOC 35813 a.

########## Paratypes.

CHIOC 35813 b (allotype ♀), 35813 c (♂), 35813 d (2♀♀).

########## Remarks.

One paratype male and two females deposited in the IPCAS collection.

########## Reference.

Vieira et al. (2012).

####### Cucullanidae Cobbold, 1864

######## *Cucullanus* Mueller, 1777

######### Cucullanus ageneiosi

Taxon classificationAnimaliaRhabditidaCucullanidae

Giese, Lanfredi & Santos, 2010

########## Type host.

*Ageneiosus ucayalensis*

########## Infection site.

Small intestine.

########## Type locality.

Brazil, Pará State, Belém, Guajará Bay (1°15'–1°29'S, 48°32–48°29'W).

########## Holotype.

♂ CHIOC 35657 a.

########## Paratype.

CHIOC 35657 b (allotype♀).

########## Reference.

Giese et al. (2010).

######### Cucullanus brevicaudatus

Taxon classificationAnimaliaRhabditidaCucullanidae

Pereira, Vieira & Luque, 2014

########## Type host.

*Balistes capriscus* Gmelin, 1789 (Osteichthyes: Balistidae).

########## Infection site.

Intestine.

########## Type locality.

Brazil, Rio de Janeiro State, Angra dos Reis Bay (23°00'S, 44°–10'W).

########## Holotype.

♂ CHIOC 35896 a.

########## Paratypes.

CHIOC 35896 b (allotype ♀), 35897 (3♂♂, 3♀♀).

########## Remarks.

Two paratypes males and two females deposited in the IPCAS collection.

########## Reference.

Pereira et al. (2014c).

######### Cucullanus brevispiculus

Taxon classificationAnimaliaRhabditidaCucullanidae

Moravec, Kohn & Fernandes, 1993

########## Type host.

*Auchenipterus nuchalis* (Spix & Agassiz, 1829) (Osteichthyes: Auchenipteridae).

########## Infection site.

Intestine.

########## Type locality.

Brazil, Paraná State, Foz do Iguaçú, Reservoir of the hydroelectric power station of Itaipú.

########## Holotype.

♂ CHIOC 32951.

########## Remarks.

One specimen deposited, no paratypes.

########## Reference.

Moravec et al. (1993).

######### Cucullunus cassinensis

Taxon classificationAnimaliaRhabditidaCucullanidae

Pereira Jr. & Costa, 1996

########## Type host.

*Micropogonias furnieri* (Desmarest, 1823) (Osteichthyes: Sciaenidae).

########## Infection site.

Digestive tract.

########## Type locality.

Brazil, Rio Grande do Sul State, Rio Grande.

########## Holotype.

♀ CHIOC 33655.

########## Paratype.

CHIOC 33656 (allotype ♂).

########## Remarks.

Other paratypes deposited in CHIP-URG.

########## Reference.

Pereira Jr. and Costa (1996).

######### Cucullanus gastrophysi

Taxon classificationAnimaliaRhabditidaCucullanidae

Pereira, Vieira & Luque, 2015 in Vieira et al. (2015)

########## Type host.

*Lophius gastrophysus* Miranda-Ribeiro, 1915 (Osteichthyes: Lophiidae).

########## Infection site.

Intestine.

########## Type locality.

Brazil, Rio de Janeiro State, Cabo Frio (22°52' S, 42°01' W).

########## Holotype.

♂ CHIOC 36745 a.

########## Paratypes.

CHIOC 36745 b (allotype ♀), 36746 (2♂♂, 2♀♀).

########## Remarks.

One paratype male and one female deposited in the IPCAS collection.

########## Reference.

Vieira et al. (2015).

######### Cucullanus protudens

Taxon classificationAnimaliaRhabditidaCucullanidae

Pereira, Vieira & Luque, 2015 in Vieira et al. (2015)

########## Type host.

*Pagrus pagrus* (Linnaeus, 1758) (Osteichthyes: Sparidae).

########## Infection site.

Intestine.

########## Type locality.

Brazil, Rio de Janeiro State, Cabo Frio (22°52'S, 42°01'W).

########## Holotype.

♂ CHIOC 36747 a.

########## Paratypes.

CHIOC 36747 b (allotype ♀), 36748 (♀).

########## Reference.

Vieira et al. (2015).

######### Cucullanus pseudopercis

Taxon classificationAnimaliaRhabditidaCucullanidae

Pereira, Vieira & Luque, 2015 in Vieira et al. (2015)

########## Type host.

*Pseudopercis semifasciata* (Cuvier, 1829)

########## Infection site.

Intestine.

########## Type locality.

Brazil, Rio de Janeiro State, Cabo Frio (22°52' S, 42°01' W).

########## Holotype.

♂ CHIOC 36749 a.

########## Paratypes.

CHIOC 36749 b (allotype ♀), 36750 (♂, ♀).

########## Remarks.

One paratype male deposited in the IPCAS collection.

########## Reference.

Vieira et al. (2015).

######### Cucullanus grandistomis

Taxon classificationAnimaliaRhabditidaCucullanidae

(Ferraz & Thatcher, 1988)

########## Type host.

*Oxydoras niger* (Valenciennes, 1821) (Osteichthyes: Doradidae).

########## Infection site.

Intestine.

########## Type locality.

Brazil, Amazonas State, Solimões River near Manaus.

########## Paratypes.

CHIOC 32456 a–b (♂, ♀).

########## Remarks.

Holotype and allotype deposited in the INPA collection. CHIOC numbers were not included in the original description.

########## Reference.

Ferraz and Thatcher (1988).

######### Cucullanus heliomartinsi

Taxon classificationAnimaliaRhabditidaCucullanidae

Moreira, Rocha & Costa, 2000

########## Type host.

*Trachelyopterus striatulus* (Steindachner, 1877) [= *Parauchenipterus striatulus*] (Osteichthyes: Auchenipteridae).

########## Infection site.

Intestine.

########## Type locality.

Brazil, Minas Gerais State, Central Lake of the State Park of Rio Doce (19°50'S, 42°35'W).

########## Holotype.

♂ CHIOC 33863 a.

########## Paratype.

CHIOC 33863 b (allotype ♀).

########## Reference.

Moreira et al. (2000).

######### Cucullanus pimelodellae

Taxon classificationAnimaliaRhabditidaCucullanidae

Moravec, Kohn & Fernandes, 1993

########## Type host.

*Pimelodella lateristriga* (Lichtenstein, 1823) (Osteichthyes: Heptapteridae).

########## Infection site.

Intestine.

########## Type locality.

Brazil, Paraná State, Guaíra.

########## Paratype.

CHIOC 32826 (♂).

########## Remarks.

Holotype deposited in the ASCR collection.

########## Reference.

Moravec et al. (1993).

######### Cucullanus pinnai pterodorasi

Taxon classificationAnimaliaRhabditidaCucullanidae

Moravec, Kohn & Fernandes, 1997

########## Type host.

*Pterodoras granulosus* (Valenciennes, 1821) (Osteichthyes: Doradidae).

########## Infection site.

Intestine.

########## Type locality.

Brazil, Paraná State, Guaíra (Paraná River basin), Reservoir of Itaipú.

########## Holotype

♂, **allotype** ♀ **and paratype.** CHIOC 33532.

########## Remarks.

Paratype from CHIOC cited as “33697” in the original description due to a mistake. Other paratypes deposited in the ASCR collection.

########## Reference.

Moravec et al. (1997).

######### Cucullanus pseudoplatystomae

Taxon classificationAnimaliaRhabditidaCucullanidae

Moravec, Kohn & Fernandes, 1993

########## Type host.

*Pseudoplatystoma corruscans* (Spix & Agassiz, 1829) (Osteichthyes: Pimelodidae).

########## Infection site.

Intestine.

########## Type locality.

Brazil, Paraná State, Paraná River, Guaíra.

########## Holotype.

♂ CHIOC 32829 a.

########## Paratypes.

CHIOC 32829 b (allotype ♀), 32829 c–d, 32910, 32913, 32914.

########## Remarks.

Other paratypes deposited in the ASCR collection.

########## Reference.

Moravec et al. (1993).

######### Cucullanus rhamphichthydis

Taxon classificationAnimaliaRhabditidaCucullanidae

Moravec, Kohn & Fernandes, 1997

########## Type host.

*Rhamphichthys rostratus* (Linnaeus, 1766) (Osteichthyes: Rhamphichthyidae).

########## Infection site.

Intestine.

########## Type locality.

Brazil, Paraná State, Santa Helena, reservoir of the hydroelectric power station of Itaipú.

########## Holotype.

♀ CHIOC 33533.

########## Paratypes.

CHIOC 33533 (2♀♀)

########## Remarks.

Type material from CHIOC cited as “33716” in the original description due to a mistake. Other paratypes deposited in the ASCR collection.

########## Reference.

Moravec et al. (1997).

######### Cucullanus tucunarensis

Taxon classificationAnimaliaRhabditidaCucullanidae

Lacerda, Takemoto, Marchiori, Martins & Pavanelli, 2013

########## Type host.

*Cichla piquiti* Kullander & Ferreira, 2006 (Osteichthyes: Cichlidae).

########## Infection site.

Intestine.

########## Type locality.

Brazil, Tocantins State, Lajeado reservoir (10°66'55"S, 48°42'36"W).

########## Holotype.

♀ CHIOC 35877 a.

########## Paratype.

CHIOC 35877 b (allotype ♂).

########## Remarks.

Other paratypes deposited in CNHE.

########## Reference.

Lacerda et al. (2013).

######## Dichelyne (Cucullanellus) (Tornquist, 1931)

######### Dichelyne (Cucullanellus) sciaenidicola

Taxon classificationAnimaliaRhabditidaCucullanidae

Timi, Lanfranchi, Tavares & Luque, 2009

########## Type host.

*Umbrina canosai* Berg, 1895 (Osteichthyes: Sciaenidae).

########## Infection site.

Posterior end of intestine.

########## Type locality.

Argentina, Buenos Aires Province, Mar del Plata (38°08'S, 57°32'W).

########## Paratypes.

CHIOC 35615 (5♂♂), 35616 (2♀♀), 35617 (5♂♂), 35618 (5♀♀).

########## Remarks.

CHIOC 35617 and 35618 collected in Pedra de Guaratiba (23°01'S, 43°38'W), Rio de Janeiro (Rio de Janeiro State, Brazil) from the sciaenid fish *Micropogonias furnieri*. Holotype, allotype, and other paratypes deposited in CHMLP.

########## Reference.

Timi et al. (2009).

######### Dichelyne (Cucullanellus) tornquisti

Taxon classificationAnimaliaRhabditidaCucullanidae

Paschoal, Vieira, Cezar & Luque, 2014

########## Type host.

*Orthopristis ruber* (Cuvier, 1830) (Osteichthyes: Haemulidae).

########## Infection site.

Intestine.

########## Type locality.

Brazil, coast of the Rio de Janeiro State (21–23°S, 42–45°W).

########## Holotype.

♂ CHIOC 35894 a.

########## Paratypes.

CHIOC 35894 b (allotype ♀), 35895 (2♂♂, 2♀♀).

########## Remarks.

One paratype male and two females deposited in the IPCAS collection.

########## Reference.

Paschoal et al. (2014).

######## Dichelyne (Dichelyne) Jägerskiöld, 1902

######### Dichelyne (Dichelyne) pimelodi

Taxon classificationAnimaliaRhabditidaCucullanidae

Moravec, Kohn & Fernandes, 1997

########## Type host.

*Pimelodus maculatus*

########## Infection site.

Intestine.

########## Type locality.

Brazil, Paraná State, Guaíra (Paraná River basin), Reservoir of Itaipú.

########## Holotype.

♂ CHIOC 33534.

########## Remarks.

Holotype cited as “33717” in the original description due to a mistake.

########## Reference.

Moravec et al. (1997).

######### Dichelyne (Dichelyne) micropogonii

Taxon classificationAnimaliaRhabditidaCucullanidae

Pereira Jr. & Costa, 1996

########## Type host.

*Micropogonias furnieri*

########## Infection site.

Digestive tract.

########## Type locality.

Brazil, Rio Grande do Sul State, Rio Grande.

########## Holotype.

♀ CHIOC 33649.

########## Paratypes.

CHIOC 33650 (allotype ♂), 33651 (♀), 33652 (♀), 33653 a–b (♂♂), 33654 (♂).

########## Remarks.

Other paratypes deposited in CHIP-URG.

########## Reference.

Pereira Jr. and Costa (1996).

####### Cystidicolidae Skrjabin, 1946

######## *Ascarophis* van Beneden, 1871

######### Ascarophis brasiliensis

Taxon classificationAnimaliaRhabditidaCystidicolidae

Pinto, Vicente & Noronha, 1984

########## Type host.

*Trachinotus carolinus* (Linnaeus, 1766) (Osteichthyes: Carangidae).

########## Infection site.

Stomach.

########## Type locality.

Brazil, Rio de Janeiro State, Araruama (22°52'23"S, 42°20'20"W).

########## Holotype.

♂ CHIOC 32032 a.

########## Paratypes.

CHIOC 32032 b–d, f, h (♀♀), e, g (♂♂), 32033 a–b (♀, ♂).

########## Reference.

Pinto et al. (1984).

######## *Comephoronema* Layman, 1933

######### Comephoronema multipapillatum

Taxon classificationAnimaliaRhabditidaCystidicolidae

Pereira, Pereira & Luque, 2014

########## Type host.

*Holocentrus adscensionis* (Osbeck, 1765) (Osteichthyes: Holocentridae).

########## Infection site.

Anterior intestine and caecum.

########## Type locality.

Brazil, Rio de Janeiro State, Angra dos Reis Bay.

########## Holotype.

♂ CHIOC 35879 a.

########## Paratypes.

CHIOC 35879 b (allotype ♀), 35880 (♀), 35881 (♂), 35882 (♂).

########## Remarks.

Two paratypes males and one female deposited in the IPCAS collection.

########## Reference.

Pereira et al. (2014b).

######## *Neoascarophis* Skrjabin, 1946

######### Neoascarophis mariae

Taxon classificationAnimaliaRhabditidaCystidicolidae

Pereira, Timi, Vieira & Luque, 2012

########## Type host.

*Mullus argentinae* Hubbs & Marini, 1933 (Osteichthyes: Mullidae).

########## Infection site.

Stomach and intestine.

########## Type locality.

Brazil, Rio de Janeiro State, Rio de Janeiro (22°55'S, 43°12'W).

########## Holotype.

♂ CHIOC 35802 a.

########## Paratypes.

CHIOC 35802 b (15♀♀), 35803 a (allotype ♀), 35803 b (12♂♂).

########## Remarks.

Other paratypes deposited in the IPCAS collection.

########## Reference.

Pereira et al. (2012a).

######## *Pseudascarophis* Ko, Margolis & Machida, 1985

######### Pseudascarophis brasiliensis

Taxon classificationAnimaliaRhabditidaCystidicolidae

Pereira, Pereira, Timi & Luque, 2013

########## Type host.

*Kyphosus sectatrix* (Linnaeus, 1758) (Osteichthyes: Kyphosidae).

########## Infection site.

Stomach.

########## Type locality.

Brazil, Rio de Janeiro State, Angra dos Reis (23°00'S, 44°10'W).

########## Holotype.

♂ CHIOC 35848 a.

########## Paratypes.

CHIOC 35848 b (allotype ♀), 35849 a–b (2♂♂, 2♀♀).

########## Reference.

Pereira et al. (2013).

####### Diplotriaenidae Skrjabin, 1916

######## *Diplotriaena* Railliet & Henry, 1909

######### Diplotriaena zederi

Taxon classificationAnimaliaRhabditidaDiplotriaenidae

Pinto, Vicente & Noronha, 1981

########## Type host.

*Xiphocolaptes albicollis* (Vieillot, 1818) (Aves: Dendrocolaptidae).

########## Infection site.

Body cavity.

########## Type locality.

Brazil, Rio de Janeiro State, Angra dos Reis.

########## Holotype.

♂ CHIOC 31826 a.

########## Paratypes.

CHIOC 31826 b–d, g (♂♂), e–f, h (♀♀), 31827 a (♂), b–c (♀♀).

########## Reference.

Pinto et al. (1981).

####### Dracunculidae Stiles, 1907

######## *Dracunculus* Reichard, 1759

######### Dracunculus brasiliensis

Taxon classificationAnimaliaRhabditidaDracunculidae

Moravec & Santos, 2009

########## Type host.

*Eunectes murinus* (Linnaeus, 1758) (Serpentes: Boidae).

########## Infection site.

Subcutaneous tissue (inside skin swelling) and body cavity (mesentery near lung).

########## Type locality.

Brazil, Pará State, Mexiana Island (Amazon River delta), Santo Ambrósio Farm (00°05'30"S, 49°34'50"W).

########## Holotype.

♀ CHIOC 35610 a.

########## Remarks.

Paratype deposited in the IPCAS collection.

########## Reference.

Moravec and Santos (2009).

####### Habronematidae Chitwood & Wehr, 1932

######## *Procyrnea* Chabaud, 1975

######### Procyrnea anterovulvata

Taxon classificationAnimaliaRhabditidaHabronematidae

Pinto, Vicente & Noronha, 1996

########## Type host.

*Chelidoptera tenebrosa brasiliensis* Sclater, 1862 (Aves: Bucconidae).

########## Infection site.

Gizzard.

########## Type locality.

Brazil, Espírito Santo State, Conceição da Barra.

########## Holotype.

♂ CHIOC 32783 a.

########## Paratypes.

CHIOC 32783 b (♂), 32783 c, e–f (♀♀), 32783 d (allotype ♀).

########## Reference.

Pinto et al. (1996).

####### Heligmonellidae Durette-Desset & Chabaud, 1977

######## *Freitastrongylus* Gonçalves, Pinto & Durette-Desset, 2007

######### * Freitastrongylus angelae

Taxon classificationAnimaliaRhabditidaHeligmonellidae

Gonçalves, Pinto & Durette-Desset, 2007

########## Type host.

*Dasyprocta fuliginosa* Wagler, 1832 (Rodentia: Dasyproctidae).

########## Infection site.

Stomach.

########## Type locality.

Brazil, Amazonas State, Barcelos, Jauari waterway, left margin of the Aracá River.

########## Holotype.

♂ CHIOC 35044 a.

########## Paratypes.

CHIOC 34844, 35044 b (allotype ♀), c–f (♀♀), g–k (♂♂), l–m (synlophe ♂♂), n (synlophe).

########## Remarks.

*Dasyprocta leporina* (Linnaeus, 1758) was cited as the type host in the original description, but *D. fuliginosa* is the host of all type material of CHIOC as indicated in the cataloguing data. Other male and female paratypes deposited in the MNHN collection.

########## Reference.

Gonçalves et al. (2007).

######## *Guerrerostrongylus* Sutton & Durette-Desset, 1991

######### Guerrerostrongylus gomesae

Taxon classificationAnimaliaRhabditidaHeligmonellidae

Simões, Santos & Maldonado Jr., 2012

########## Type host.

*Oecomys mamorae* (Thomas, 1906) (Rodentia: Cricetidae).

########## Infection site.

Small intestine.

########## Type locality.

Brazil, Mato Grosso do Sul State, Rio Negro Farm, Aquidauana (19°34'54"S, 56°14'62"W).

########## Holotype.

♂ CHIOC 37233.

########## Paratypes.

CHIOC 37234 (allotype ♀), 35667 (10♂♂, 6♀♀).

########## Reference.

Simões et al. (2012).

######### Guerrerostrongylus marginalis

Taxon classificationAnimaliaRhabditidaHeligmonellidae

Weirich, Catzeflis & Jiménez, 2016

########## Type host.

*Oecomys auyantepui* Tate, 1939

########## Infection site.

Small intestine.

########## Type locality.

French Guiana, Municipality of Roura, Cacao (04°33'708"N, 52°26'590"W), 197 m high.

########## Paratypes.

CHIOC 38104, 38105.

########## Remarks.

Holotype, allotype and other paratypes deposited in the MNHN collection. Additional paratypes deposited in CNHE and HWML.

########## Reference.

Weirich et al. (2016).

######## *Hassalstrongylus* Durette-Desset, 1971

######### Hassalstrongylus luquei

Taxon classificationAnimaliaRhabditidaHeligmonellidae

Costa, Maldonado Jr., Bóia, Lucio & Simões, 2014

########## Type host.

*Euryoryzomys russatus* (Wagner, 1848) (Rodentia: Cricetidae).

########## Infection site.

Small intestine.

########## Type locality.

Brazil, Rio de Janeiro State, Angra dos Reis, Ilha Grande (23°09'07.50"S, 44°13'44.20"W).

########## Holotype.

♂ CHIOC 35928 a.

########## Paratypes.

CHIOC 35928 b–c (7♂♂).

########## Reference.

Costa et al. (2014).

######## *Stilestrongylus* Freitas, Lent & Almeida, 1937

######### Stilestrongylus lanfrediae

Taxon classificationAnimaliaRhabditidaHeligmonellidae

Souza, Digiani, Simões, Luque, Rodrigues-Silva & Maldonado Jr., 2009

########## Type host.

*Oligoryzomys nigripes*

########## Infection site.

Small intestine.

########## Type locality.

Brazil, Rio de Janeiro State, Serra dos Órgãos, Teresópolis (22°12'44"S, 42°48'40"W).

########## Holotype.

♂ CHIOC 36925 a.

########## Paratypes.

CHIOC 35536 (10♂♂, 10♀♀), 36925 b (allotype ♀).

########## Reference.

Souza et al. (2009).

####### Heterakidae Railliet & Henry, 1912

######## *Africana* Travassos, 1920

######### Africana dardanelosi

Taxon classificationAnimaliaRhabditidaHeterakidae

Ávila & Silva, 2009

########## Type host.

*Hoplocercus spinosus* Fitzinger, 1843 (Iguania: Hoplocercidae).

########## Infection site.

Small and large intestine.

########## Type locality.

Brazil, Mato Grosso State, Aripuanã (10°9'0"S, 59°27'0"W).

########## Holotype.

♂ CHIOC 35614 a.

########## Paratype.

CHIOC 35614 b (allotype ♀).

########## Remarks.

Holotype and allotype cited as “CHIOC 3561” in the original description due to a mistake. Other paratypes deposited in CHIBB.

########## Reference.

Ávila and Silva (2009).

######## *Heterakis* Dujardin, 1845

######### Heterakis inglisi

Taxon classificationAnimaliaRhabditidaHeterakidae

Vicente, Pinto & Noronha, 1993

########## Type host.

*Crypturellus variegatus variegatus* (Gmelin, 1789) (Aves: Tinamidae).

########## Infection site.

Intestine.

########## Type locality.

Brazil, Espírito Santo State.

########## Holotype.

♂ CHIOC 32850 a.

########## Paratypes.

CHIOC 32850 b–e (♂♂), f–j (♀♀), 32851 a–d (♂♂).

########## Reference.

Vicente et al. (1993).

####### Hystrignathidae Travassos, 1919

######## *Artigasia* Christie, 1934

######### Artigasia milerai

Taxon classificationAnimaliaRhabditidaHystrignathidae

Morffe & García, 2010

########## Type host.

*Passalus interstitialis* Eschischoltz, 1829 (Coleoptera: Passalidae).

########## Infection site.

Gut caeca.

########## Type locality.

Cuba, La Habana province, Jaruco, Escaleras de Jaruco.

########## Paratypes.

CHIOC 37823 (2♀♀).

########## Remarks.

CHIOC number was not included in the original description. Holotype female and additional paratypes females deposited in CZACC.

########## Reference.

Morffe and García (2010a).

######## *Hystrignatus* Leidy, 1850

######### Hystrignatus dearmasi

Taxon classificationAnimaliaRhabditidaHystrignathidae

Morffe & García, 2010

########## Type host.

Unidentified, short, blackish passalid beetle (Coleoptera: Passalidae).

########## Infection site.

Gut caeca.

########## Type locality.

Panama, Panama Province, Summit National Park.

########## Paratypes.

CHIOC 37525 (2♀♀).

########## Remarks.

CHIOC number was not included in the original description. Holotype female and additional paratypes females deposited in CZACC.

########## Reference.

Morffe and García (2010b).

######## *Batwanema* Morffe & García, 2013

######### * Batwanema congo

Taxon classificationAnimaliaRhabditidaHystrignathidae

Morffe & García, 2013

########## Type host.

*Pentalobus barbatus* (Fabricius, 1801) (Coleoptera: Passalidae).

########## Infection site.

Gut caeca.

########## Type locality.

Democratic Republic of Congo, Ituri province, Mogwalu.

########## Paratype.

CHIOC 37903 (♀).

########## Remarks.

CHIOC number was not included in the original description. Holotype and paratype females deposited in CZACC. Other paratypes deposited in the RMCA collection.

########## Reference.

Morffe and García (2013a).

######## *Chokwenema* Morffe & García, 2013

######### * Chokwenema lepidophorum

Taxon classificationAnimaliaRhabditidaHystrignathidae

Morffe & García, 2013

########## Type host.

Didimoides cf. parastictus (Imhoff, 1843) (Coleoptera: Passalidae).

########## Infection site.

Gut caeca.

########## Type locality.

Democratic Republic of Congo, Ituri province, Mogwalu.

########## Paratype.

CHIOC 37904 (♀).

########## Remarks.

CHIOC number was not included in the original description. Holotype and paratype females deposited in CZACC. Other paratypes deposited in the RMCA collection.

########## Reference.

Morffe and García (2013a).

######## *Kongonema* Morffe & García, 2013

######### * Kongonema meyeri

Taxon classificationAnimaliaRhabditidaHystrignathidae

Morffe & García, 2013

########## Type host.

*Didimus* sp. (Coleoptera: Passalidae).

########## Infection site.

Gut caeca.

########## Type locality.

Democratic Republic of Congo, Kivu Region, Katale (01°19'S, 29°22'E).

########## Paratypes.

CHIOC 37822 a–d (♂♂).

########## Remarks.

CHIOC number was not included in the original description. Female holotype and male and female paratypes deposited in CZACC. Other paratypes deposited in the RMCA collection.

########## Reference.

Morffe and García (2013b).

####### Metastrongylidae Leiper, 1908

######## *Angiostrongylus* Kamensky, 1905

######### Angiostrongylus felineus

Taxon classificationAnimaliaRhabditidaMetastrongylidae

Vieira, Muniz-Pereira, Lima, Moraes Neto, Guimarães & Luque, 2013

########## Type host.

*Puma yagouaroundi* (Geoffroy, 1803) (Carnivora: Felidae).

########## Infection site.

Pulmonary arteries.

########## Type locality.

Brazil, Minas Gerais State, Juiz de Fora (21°41'20"S, 43°20'40"W).

########## Holotype.

♂ CHIOC 35812 a.

########## Paratypes.

CHIOC 35812 b (allotype ♀), 35812 c–d (♂, ♀).

########## Reference.

Vieira et al. (2013).

####### Molineidae Durette-Desset & Chabaud, 1977

######## *Hadrostrongylus* Hoppe & Nascimento, 2007

######### * Hadrostrongylus speciosum

Taxon classificationAnimaliaRhabditidaMolineidae

Hoppe & Nascimento, 2007

########## Type host.

*Dasypus novemcinctus*

########## Infection site.

Mucosa and lumen of the cecum and colon.

########## Type locality.

Brazil, Mato Grosso do Sul State, Aquidauana.

########## Paratypes.

CHIOC 35448 (2♂♂, 2♀♀).

########## Remarks.

Male holotype and female allotype deposited in the FCAV/UNESP helminthological collection of Jaboticabal, São Paulo State.

########## Reference.

Hoppe and Nascimento (2007).

######## *Oswaldocruzia* Travassos, 1917

######### Oswaldocruzia belenensis

Taxon classificationAnimaliaRhabditidaMolineidae

Santos, Geise, Maldonado & Lanfredi, 2008

########## Type host.

*Rhinella marina* (Linnaeus, 1758) [= *Chaunus marinus*] (Amphibia: Bufonidae).

########## Infection site.

Small intestine.

########## Type locality.

Brazil, Pará State, Belém (01°28'03"S, 48°20'18"W).

########## Holotype.

CHIOC 36855 a.

########## Paratypes.

CHIOC 36855 b (allotype ♀), 36855 c, f (♀♀), d–e (♂♂).

########## Reference.

Santos et al. (2008b).

######## *Schulzia* Travassos, 1937

######### Schulzia usu

Taxon classificationAnimaliaRhabditidaMolineidae

Lent & Santos, 1989

########## Type host.

*Atelopus oxyrhynchus*

########## Infection site.

Small intestine.

########## Type locality.

Venezuela, Mérida.

########## Holotype

♂ **and allotype** ♀. CHIOC 31668.

########## Paratype.

CHIOC 32355.

########## Reference.

Lent and Santos (1989).

####### Onchocercidae Leiper, 1911

######## *Cercopithifilaria* Eberhard, 1980

######### Cercopithifilaria bainae

Taxon classificationAnimaliaRhabditidaOnchocercidae

Almeida & Vicente, 1984

########## Type host.

*Canis familiaris* Linnaeus, 1758 (Carnivora: Canidae).

########## Infection site.

Subcutaneous and intramuscular tissue.

########## Type locality.

Brazil, Rio de Janeiro State, Rio de Janeiro.

########## Holotype.

♂ CHIOC 32176 a.

########## Paratype.

CHIOC 32176 b (allotype ♀).

########## Reference.

Almeida and Vicente (1984).

######## *Litomosoides* Chandler, 1931

######### Litomosoides chagasfilhoi

Taxon classificationAnimaliaRhabditidaOnchocercidae

Moraes Neto, Lanfredi & Souza, 1997

########## Type host.

*Akodon cursor* (Winge, 1887) (Rodentia: Muridae).

########## Infection site.

Abdominal cavity.

########## Type locality.

Brazil, Rio de Janeiro State, Rio Bonito, Catimbau Grande (22°42'30"S, 42°37'34"W).

########## Holotype.

♂ CHIOC 33529.

########## Paratype.

CHIOC 33530 (allotype ♀).

########## Remarks.

Other paratypes deposited in the MNHN collection.

########## Reference.

Moraes Neto et al. (1997).

######### Litomosoides galizai

Taxon classificationAnimaliaRhabditidaOnchocercidae

Bain, Petit & Diagne, 1989

########## Type host.

*Oecomys trinitatis* (Allen & Chapman, 1893)

########## Infection site.

Body cavity and lung.

########## Type locality.

Brazil, Pará State, Carajás.

########## Paratypes.

CHIOC 32643 (2♀♀).

########## Remarks.

Holotype and allotype deposited in the MNHN collection. CHIOC number was not included in the original description.

########## Reference.

Bain et al. (1989).

######### Litomosoides kohnae

Taxon classificationAnimaliaRhabditidaOnchocercidae

Bain, Petit & Diagne, 1989

########## Type host.

*Nectomys squamipes*

########## Infection site.

Body cavity and lung.

########## Type locality.

Brazil, São Paulo State, São Paulo.

########## Holotype.

♀ CHIOC 31056 c.

########## Paratypes.

CHIOC 31056 a (♂), 31056 b (allotype ♂), 32642 (♀, = 31056 d).

########## Remarks.

The slide CHIOC 31056 d was dismounted and received the number 32642.

########## Reference.

Bain et al. (1989).

######## *Ornithofilaria* Gonnert, 1937

######### Ornithofilaria pitangi

Taxon classificationAnimaliaRhabditidaOnchocercidae

Vicente, Pinto & Noronha, 1980

########## Type host.

*Megarynchus pitangua* (Linnaeus, 1766) (Aves: Tyrannidae).

########## Infection site.

Infraorbital sinus.

########## Type locality.

Brazil, Rio de Janeiro State, Angra dos Reis.

########## Holotype.

♂ CHIOC 31782 a.

########## Paratypes.

CHIOC 31782 b–e (♂♂), f–j (♀♀).

########## Reference.

Vicente et al. (1980).

####### Oxyuridae Cobbold, 1864

######## *Caroloxyuris* Jiménez-Ruiz & Gardner, 2003

######### * Caroloxyuris boliviensis

Taxon classificationAnimaliaRhabditidaOxyuridae

Jiménez-Ruiz & Gardner, 2003

########## Type host.

*Oxymycterus paramensis* Thomas, 1902 (Rodentia: Cricetidae).

########## Infection site.

Cecum and large intestine.

########## Type locality.

Bolivia, Chuquisaca Department, 2 km SW of Monteagudo (19°49'S, 63°58'W), 1130 m high.

########## Paratype.

CHIOC 34809.

########## Remarks.

Paratype from CHIOC collected in Cochabamba, 13 km N of Colomi (17°13'29"S, 65°53"30"W), 3152 m high. Holotype, allotype, and paratypes deposited in the HWML collection. Other two paratypes deposited in CNHE.

########## Reference.

Jiménez-Ruiz and Gardner (2003).

######## *Gracilioxyuris* Feijó, Lopes Torres, Maldonado Jr. & Lanfredi, 2008

######### * Gracilioxyuris agilis

Taxon classificationAnimaliaRhabditidaOxyuridae

Feijó, Torres, Maldonado Jr. & Lanfredi, 2008

########## Type host.

*Gracilinanus agilis* (Burmeister, 1854) (Didelphimorphia: Didelphidae).

########## Infection site.

Cecum.

########## Type locality.

Brazil, Mato Grosso do Sul State, Pantanal, Nhumirim Farm (18°59'00"S, 56°39'00"W).

########## Holotype.

♂ 35518 a.

########## Paratypes.

CHIOC 35517 (allotype ♀), 35518 b.

########## Remarks.

CHIOC 35517 was collected in the Rio Negro Farm, Nhecolândia Region (19°34'54"S, 56°14'62"W), Pantanal, Mato Grosso do Sul State.

########## Reference.

Feijó et al. (2008).

####### Pharyngodonidae Travassos, 1919

######## *Brasilnema* Moravec, Kohn & Fernandes, 1992

######### * Brasilnema pimelodellae

Taxon classificationAnimaliaRhabditidaPharyngodonidae

Moravec, Kohn & Fernandes, 1992

########## Type host.

*Pimelodella lateristriga*

########## Infection site.

Intestine.

########## Type locality.

Brazil, Paraná State, Paraná River, Guaíra.

########## Paratypes.

CHIOC 32718 a–b, e (♂♂), c–d, f–i (♀♀), 32719 a–d (♀♀).

########## Remarks.

Holotype, allotype, and other paratypes deposited in the IPCAS collection. Additional paratypes deposited in MNHN.

########## Reference.

Moravec et al. (1992a).

######## *Ichthyouris* Inglis, 1962

######### Ichthyouris brasiliensis

Taxon classificationAnimaliaRhabditidaPharyngodonidae

Moravec, Kohn & Fernandes, 1992

########## Type host.

*Megalancistrus parananus* (Peters, 1881) (Osteichthyes: Locariidae).

########## Infection site.

Intestine.

########## Type locality.

Brazil, Paraná State, Paraná River, Guaíra.

########## Paratypes.

CHIOC 32649, 32721 a–b (♂, ♀).

########## Remarks.

Holotype, allotype, and other paratypes deposited in the IPCAS collection. Additional paratypes deposited in MNHN.

########## Reference.

Moravec et al. (1992a).

######### Ichthyouris laterifilamenta

Taxon classificationAnimaliaRhabditidaPharyngodonidae

Moravec, Kohn & Fernandes, 1992

########## Type host.

*Trachydoras paraguayensis* (Eigenmann & Ward, 1907) (Osteichthyes: Doradidae).

########## Infection site.

Intestine.

########## Type locality.

Brazil, Paraná State, Foz do Iguaçú, Reservoir of the hydroelectric power of Itaipú (24°20'S, 52°38'W).

########## Paratype.

CHIOC 32925.

########## Remarks.

Holotype, allotype, and other paratypes deposited in the IPCAS collection.

########## Reference.

Moravec et al. (1992b).

######### Ichthyouris voltagrandensis

Taxon classificationAnimaliaRhabditidaPharyngodonidae

Martins, Yoshitoshi & Umekita, 2001

########## Type host.

*Myleus tiete* (Eigenmann & Norris, 1900) (Osteichthyes: Serrasalmidae).

########## Infection site.

Intestine.

########## Type locality.

Brazil, Minas Gerais State, Volta Grande Reservoir.

########## Holotype.

♂ CHIOC 33852 a.

########## Paratypes.

CHIOC 33852 b (allotype ♀), 33852 c (5♂♂), 33852 d (7♀♀).

########## Reference.

Martins et al. (2001).

######## *Parasynodontisia* Moravec, Kohn & Fernandes, 1992

######### * Parasynodontisia petterae

Taxon classificationAnimaliaRhabditidaPharyngodonidae

Moravec, Kohn & Fernandes, 1992

########## Type host.

*Rhinelepis aspera* Spix & Agassiz, 1829 (Osteichthyes: Locariidae).

########## Infection site.

Intestine.

########## Type locality.

Brazil, Paraná State, Paraná River, Guaíra.

########## Paratypes.

CHIOC 32652, 32720 a (♂), b (♀).

########## Remarks.

Holotype, allotype, and other paratypes deposited in the IPCAS collection. Additional paratypes deposited in MNHN.

########## Reference.

Moravec et al. (1992b).

######## *Skrjabinodon* Inglis, 1968

######### Skrjabinodon heliocostai

Taxon classificationAnimaliaRhabditidaPharyngodonidae

Vicente, Vrcibradic, Muniz-Pereira & Pinto, 2000

########## Type host.

*Notomabuya frenata* (Cope, 1862) [= *Mabuya frenata*] (Autarchoglossa: Scincidae).

########## Infection site.

Large intestine.

########## Type locality.

Brazil, São Paulo State, Valinhos.

########## Holotype

. ♂ CHIOC 33965 a.

########## Paratypes

. CHIOC 33965 b (allotype ♀), CHIOC 33965 c–e (♂♂), f–h (♀♀).

########## Reference.

Vicente et al. (2000b).

######### Skrjabinodon spinosulus

Taxon classificationAnimaliaRhabditidaPharyngodonidae

Vicente, Vrcibradic, Rocha & Pinto, 2002

########## Type host.

*Aspronema dorsivittatum* (Cope, 1862) [= *Mabuya dorsivittata*] (Autarchoglossa: Scincidae).

########## Infection site.

Large and small intestine.

########## Type locality.

Brazil, Rio de Janeiro State, National Park of Itatiaia, Prateleiras.

########## Holotype.

♀ CHIOC 34539 a.

########## Paratypes.

CHIOC 34539 b–c (♀♀), 34540 a–b (♂♂), 34541 a–b (♀♀), c (♂), d (2♀♀), e–f (larvae).

########## Remarks.

Paratypes CHIOC 34541 a–f collected in the Ecological Station of Itirapina, São Paulo State.

########## Reference.

Vicente et al. (2002).

####### Philometridae Baylis & Daubney, 1926

######## *Philometra* Costa, 1845

######### Philometra nattereri

Taxon classificationAnimaliaRhabditidaPhilometridae

Cárdenas, Moravec, Fernandes & Morais, 2012

########## Type host.

*Pygocentrus nattereri* Kner, 1858 (Osteichthyes: Serrasalmidae).

########## Infection site.

Oculo-orbits and nasal mucosa.

########## Type locality.

Brazil, Amazonas State, Maracá Lake (03°50'32.8"S, 62°34'32.4"W), Coari.

########## Holotype.

♀ CHIOC 35777.

########## Paratypes.

CHIOC 35778 (2♀♀), 35779 (♀), 35780 (♀), 35781 (♀), 35782.

########## Remarks.

CHIOC 35779 collected in the Baixio Lake (03°17'27.2^"^S, 60°04'29.6"W), Iranduba; CHIOC 35780 collected in the Iauara Lake (03°36'39.2"S, 61°16'33.0"W), Manacapuru; CHIOC 35781 collected in the Araçá Lake (03°46'15.8"S, 62°20'10.3"W), Codajás; CHIOC 35782 collected in the Ananá Lake (03°53'54.8"S, 61°40'18.4"W), Anori (all localities in the Amazonas State). Other paratypes deposited in the INPA collection.

########## Reference.

Cárdenas et al. (2012).

####### Physalopteridae Railliet, 1893

######## *Physaloptera* Rudolphi, 1819

######### Physaloptera bainae

Taxon classificationAnimaliaRhabditidaPhysalopteridae

Pereira, Alves, Rocha, Souza Lima & Luque, 2014

########## Type host.

*Salvator merianae* (Duméril & Bibron, 1839) (Autarchoglossa: Teiidae).

########## Infection site.

Stomach.

########## Type locality.

Brazil, Minas Gerais State, Juiz de Fora, Parque da Lajinha (21°47'32"S, 43°22'6"W).

########## Holotype.

♂ CHIOC 35885 a.

########## Paratypes.

CHIOC 35885 b (allotype ♀), 35885 c (♂), 35885 d (2♀♀).

########## Reference.

Pereira et al. (2014b).

######### Physaloptera galvaoi

Taxon classificationAnimaliaRhabditidaPhysalopteridae

São Luiz, Simões, Lopes Torres, Barbosa, Santos, Giese, Rocha & Maldonado Jr., 2015

########## Type host.

*Cerradomys subflavus* (Wagner, 1842) (Rodentia: Cricetidae).

########## Infection site.

Stomach.

########## Type locality.

Brazil, Minas Gerais State, São Roque de Minas, Serra da Canastra National Park (20°13'28.30"S, 46°30'39.20"W).

########## Holotype.

♂ CHIOC 36758 a.

########## Paratype.

CHIOC 36758 b (allotype ♀).

########## Reference.

São Luiz et al. (2015).

######### Physaloptera herthameyerae

Taxon classificationAnimaliaRhabditidaPhysalopteridae

Lopes Torres, Maldonado Jr. & Lanfredi, 2009

########## Type host.

*Gracilinanus agilis*

########## Infection site.

Stomach.

########## Type locality.

Brazil, Mato Grosso do Sul State, Pantanal, Alegria Farm (19°15'01"S, 57°01'29"W).

########## Holotype.

♂ CHIOC 35651 a.

########## Paratypes.

CHIOC 35651 b (♂), 35652 a (allotype ♀), 36652 b (♀).

########## Reference.

Lopes Torres et al. (2009).

######### Physaloptera tupinambae

Taxon classificationAnimaliaRhabditidaPhysalopteridae

Pereira, Alves, Rocha, Lima & Luque, 2012

########## Type host.

*Salvator merianae* [= *Tupinambis merianae*]

########## Infection site.

Stomach.

########## Type locality.

Brazil, Minas Gerais State, Juiz de Fora.

########## Holotype.

♂ CHIOC 35811 a.

########## Paratypes.

CHIOC 35811 b (allotype ♂), 35811 c (♂, ♀).

########## Reference.

Pereira et al. (2012b).

####### Quimperiidae Gendre, 1928

######## *Neoparaseuratum* Moravec, Kohn & Fernandes, 1992

######### * Neoparaseuratum travassosi

Taxon classificationAnimaliaRhabditidaQuimperiidae

Moravec, Kohn & Fernandes, 1992

########## Type host.

*Pterodoras granulosus*

########## Infection site.

Intestine.

########## Type locality.

Brazil, Paraná State, Paraná River near Guaíra.

########## Holotype.

♂ CHIOC 32722 a.

########## Paratype.

CHIOC 32722 b (allotype ♀).

########## Remarks.

There is no paratype CHIOC 32722 “c” as indicated in the original description, which was a mistake. Other paratype deposited in the IPCAS collection.

########## Reference.

Moravec et al. (1992c).

####### Raphidascarididae Hartwich, 1954

######## *Hysterothylacium* Ward & Magath, 1917

######### Hysterothylacium deardorffoverstreetorum

Taxon classificationAnimaliaRhabditidaRaphidascarididae

Knoff, Felizardo, Iñiguez, Maldonado Jr., Torres, Pinto & Gomes, 2012

########## Type host.

*Paralichthys isosceles* Jordan, 1891 (Osteichthyes: Paralichthyidae).

########## Infection site.

Abdominal cavity, abdominal musculature, stomach, stomach mucosa, mesentery, intestine, heart serosa, kidney serosa, liver serosa, ovary, ovary serosa, and spleen serosa.

########## Type locality.

Brazil, Rio de Janeiro State, Angra dos Reis (21°15'–23°23'S, 40°29'–44°28'W).

########## Holotype.

CHIOC 37523 a (L3 larvae).

########## Paratypes.

CHIOC 37523 b–e, 35771 (L3 larvae).

########## Reference.

Knoff et al. (2012).

####### Rhabdiasidae Railliet, 1916

######## *Rhabdias* Stilles & Hassall, 1905

######### Rhabdias filicaudalis

Taxon classificationAnimaliaRhabditidaRhabdiasidae

Barrella, Santos & Silva, 2010

########## Type host.

*Spilotes pullatus* Linnaeus, 1758 (Serpentes: Colubridae).

########## Infection site.

Lungs.

########## Type locality.

Brazil, São Paulo State, Avaré (23°6'S, 48°55'W).

########## Holotype.

♀ CHIOC 35653 a.

########## Paratypes.

CHIOC 35653 b (5♀♀).

########## Remarks.

Other paratypes deposited in CHIBB.

########## Reference.

Barella et al. (2010).

######### Rhabdias paraensis

Taxon classificationAnimaliaRhabditidaRhabdiasidae

Santos, Melo, Silva, Giese & Furtado, 2011

########## Type host.

*Rhinella marina*

########## Infection site.

Lungs.

########## Type locality.

Brazil, Pará State, Belém (01°28'03"S, 48°20'18"W).

########## Holotype.

Hermaphrodite ♀ CHIOC 35705 a.

########## Paratypes.

CHIOC 35705 b (ten paratypes).

########## Reference.

Santos et al. (2011).

######## *Serpentirhabdias* Trach, Kuzmin & Snyder, 2014

######### Serpentirhabdias viperidicus

Taxon classificationAnimaliaRhabditidaRhabdiasidae

Morais, Aguiar, Müller, Narciso, Silva & Silva, 2016

########## Type host.

*Bothrops moojeni* Hoge, 1966

########## Infection site.

Lungs.

########## Type locality.

Brazil, São Paulo State, Castilho, Private Reserve of Natural Heritage ‘Foz do Rio Aguapeí’ (21°03'04.9"S, 51°52'52.6"W).

########## Holotype.

CHIOC 38318 a.

########## Paratype.

CHIOC 38318 b.

########## Remarks.

Other paratypes deposited in CHIBB.

########## Reference.

Morais et al. (2016).

####### Rictulariidae Hall, 1915

######## Pterygodermatites (Multipectines) Quentin, 1969

######### Pterygodermatites (Multipectines) pluripectinata

Taxon classificationAnimaliaRhabditidaRictulariidae

Hoppe, Araújo, Tebaldi & Nascimento, 2010

########## Type host.

*Cerdocyon thous*

########## Infection site.

Small intestine.

########## Type locality.

Brazil, Paraíba State, Patos (06°46'19"–07°38'52"S, 36°42'52"–38°08'56"W).

########## Paratypes.

CHIOC 35628 (♂, ♀).

########## Remarks.

Paratype from CHIOC cited as "35525" in the original description due to a mistake. Holotype and allotype deposited in the FCAV/UNESP of Jaboticabal, São Paulo State.

########## Reference.

Hoppe et al. (2010).

######## Pterygodermatites (Paucipectines) Quentin, 1969

######### Pterygodermatites (Paucipectines) andyraicola

Taxon classificationAnimaliaRhabditidaRictulariidae

Cardia, Tebaldi, Fornazari, Menozzi, Langoni, Nascimento, Bresciani & Hoppe, 2015

########## Type host.

*Eumops glaucinus* (Wagner, 1843) (Chiroptera: Molossidae).

########## Infection site.

Mucosa of the small intestine.

########## Type locality.

Brazil, São Paulo State, Botucatú (22°52'47"S, 48°26'42"W).

########## Paratypes.

CHIOC 35826 (♂, ♀).

########## Remarks.

Holotype, allotype and additional paratypes deposited in FCAV/UNESP of Araçatuba, São Paulo State. Paratypes from CHIOC cited as “35824” in the original description due to a mistake. CHIOC 35826 collected in Jaú (22°17'44"S, 48°33'28"W), São Paulo State.

########## Reference.

Cardia et al. (2015).

####### Strongyloididae Chitwood & McIntosh, 1934

######## *Strongyloides* Grass, 1879

######### Strongyloides ferrerai

Taxon classificationAnimaliaRhabditidaStrongyloididae

Rodrigues, Vicente & Gomes, 1985

########## Type host.

*Kerodon rupestris* (Wied-Neuwied, 1820) (Rodentia: Caviidae).

########## Infection site.

Small intestine.

########## Type locality.

Brazil, Piauí State, Floriano Peixoto.

########## Holotype.

♀ CHIOC 32172 a.

########## Paratypes.

CHIOC 32172 b–e (♀♀).

########## Reference.

Rodrigues et al. (1985).

####### Subuluridae Travassos, 1914

######## *Subulura* Molin, 1860

######### Subulura lacertilia

Taxon classificationAnimaliaRhabditidaSubuluridae

Vicente, Van Sluys, Fontes & Kiefer, 2000

########## Type host.

*Eurolophosaurus nanuzae* (Rodrigues, 1981) [= *Tropidurus nanuzae*] (Iguania: Tropiduridae).

########## Infection site.

Large and small intestine.

########## Type locality.

Brazil, Minas Gerais State, Serra do Cipó (19°20'S, 43°44'W).

########## Holotype.

♂ CHIOC 34196 a.

########## Paratypes.

CHIOC 34196 d (allotype ♀), 34196 b, c (♂♂), 34197 a, c, e, g (♀♀), 34197 b, d, f (♂♂), 33853, 33854 (♂), 33855 (♂), 33856 (♂).

########## Reference.

Vicente et al. (2000a).

####### Tetrameridae Travassos, 1914

######## Synhimantus (Synhimantus) Railliet, Henry & Sisoff, 1912

######### Synhimantus (Synhimantus) magnipapillatus

Taxon classificationAnimaliaRhabditidaTetrameridae

Vicente, Pinto e Noronha, 1996

########## Type host.

*Nyctanassa violacea cayennensis* (Gmelin, 1789) (Aves: Ardeidae).

########## Infection site.

Gizzard.

########## Type locality.

Brazil, Rio de Janeiro State, Rio de Janeiro.

########## Holotype.

♂ CHIOC 33182 a.

########## Paratypes.

CHIOC 33182 b, f–h (♂♂), c–e (♀♀).

########## Reference.

Vicente et al. (1996).

######## Tetrameres (Tetrameres) Creplin, 1846

######### Tetrameres (Tetrameres) spirospiculum

Taxon classificationAnimaliaRhabditidaTetrameridae

Pinto & Vicente, 1995

########## Type host.

*Theristicus caudatus caudatus* (Boddaert, 1783) (Aves: Threskiornithidae).

########## Infection site.

Gizzard (females in proventricular glands, males free in the lumen).

########## Type locality.

Brazil, Mato Grosso do Sul State, Salobra.

########## Holotype.

♂ CHIOC 33173 a.

########## Paratypes.

CHIOC 33173 b–c (♂♂), 33186 c (allotype ♀).

########## Reference.

Pinto and Vicente (1995).

####### Trichostrongylidae Witenberg, 1925

######## *Hoazinstrongylus* Pinto & Gomes, 1985

######### * Hoazinstrongylus amazonensis

Taxon classificationAnimaliaRhabditidaTrichostrongylidae

Pinto & Gomes, 1985

########## Type host.

*Opisthocomus hoazin* (Müller, 1776) (Aves: Opisthocomidae).

########## Infection site.

Proventriculus and gizzard.

########## Type locality.

Brazil, Amazonas State, Paraná de Cambixa, Careiro Island.

########## Holotype.

♂ CHIOC 32024 a.

########## Paratypes.

CHIOC 32024 b–e (♀♀), 32025 a–b (♀♀), 32026 a–b (♂♂), c–d (♀♀).

########## Reference.

Pinto and Gomes (1985).

####### Viannaiidae Durette-Desset & Chabaud, 1981

######## *Avellaria* Freitas & Lent, 1934

######### Avellaria intermedia

Taxon classificationAnimaliaRhabditidaViannaiidae

Durette-Desset, Gonçalves & Pinto, 2006

########## Type host.

*Dasyprocta fuliginosa*

########## Infection site.

Small intestine.

########## Type locality.

Brazil, Amazonas State, Barcelos, Jauari waterway, left margin of the Aracá River, Três Barracas settlement (0°58'29"S, 62°55'27"W).

########## Holotype

♂ **and allotype** ♀. CHIOC 35416.

########## Paratypes.

CHIOC 34883, 35072 a–g, 35417 (6♂♂), 35418 (7♀♀).

########## Reference.

Durette-Desset et al. (2006).

######## *Vianella* Neveu-Lemaire, 1934

######### Vianella trichospicula

Taxon classificationAnimaliaRhabditidaViannaiidae

Durette-Desset, Gonçalves & Pinto, 2006

########## Type host.

*Dasyprocta fuliginosa*

########## Infection site.

Small intestine.

########## Type locality.

Brazil, Amazonas State, Barcelos, Jauari waterway, left margin of the Aracá River, Três Barracas settlement (0°58'29"S, 62°55'27"W).

########## Holotype

♂ **and allotype** ♀. CHIOC 35419.

########## Paratypes.

CHIOC 34857 (♂, ♀), 35420 (7♂♂), 35421 (4♀♀), 35052 a–k (♂♂).

########## Reference.

Durette-Desset et al. (2006).

####### Xustrostomatidae Hunt, 2002

######## *Trachyglossoides* García & Morffe, 2015

######### * Trachyglossoides jimenoi

Taxon classificationAnimaliaRhabditidaXustrostomatidae

García & Morffe, 2015

########## Type host.

*Spirobollelus* sp. (Diplopoda: Spirobolellidae).

########## Infection site.

Hind gut.

########## Type locality.

Cuba, Mayabeque Province, San José de las Lajas, La Jaula.

########## Paratypes.

CHIOC 38211 a (♀), 38211 b (♂).

########## Remarks.

CHIOC number was not included in the original description. Male holotype and male and female paratypes deposited in CZACC. Other paratypes deposited in the RIT collection.

########## Reference.

García and Morffe (2015).

#### Phylum Cnidaria Hatschek, 1888

##### Unranked subphylum Myxozoa Grassé, 1970

###### Class Myxosporea Bütschli, 1881

####### Order Bivalvulida Shulman, 1959

######## Myxobolidae Thélohan, 1892

######### *Henneguya* Thélohan, 1892

########## Henneguya garavelli

Taxon classificationAnimaliaBivalvulidaMyxobolidae

Martins & Onaka, 2006

########### Type host.

*Cyphocharax nagelii* (Steindachner, 1881) (Osteichthyes: Curimatidae).

########### Infection site.

Gill filaments.

########### Type locality.

Brazil, São Paulo State, São José do Rio Pardo, Rio do Peixe Reservoir.

########### Holotype.

CHIOC 34986.

########### Paratype.

CHIOC 34818 (fixed gills).

########### Remarks.

CHIOC samples cited as “specimens deposited” in the original description. No additional type material is deposited in other collections.

########### Reference.

Martins and Onaka (2006).

######### *Myxobolus* Bütschli, 1882

########## Myxobolus peculiaris

Taxon classificationAnimaliaBivalvulidaMyxobolidae

Martins & Onaka, 2006

########### Type host.

*Cyphocharax nagelii*

########### Infection site.

Gill filaments.

########### Type locality.

Brazil, São Paulo State, São José do Rio Pardo, Rio do Peixe Reservoir.

########### Holotype.

CHIOC 34987.

########### Paratype.

CHIOC 34834 (fixed gills).

########### Remarks.

CHIOC samples cited as “specimens deposited” in the original description. No additional type material is deposited in other collections.

########### Reference.

Martins and Onaka (2006).

#### Phylum Annelida Lamarck, 1809

##### Class Polychaeta Grube, 1850

###### Order Eunicida Dales, 1962

####### Histriobdellidae Vaillant, 1890

######## *Stratiodrilus* Haswell, 1900

######### Stratiodrilus vilae

Taxon classificationAnimaliaEunicidaHistriobdellidae

Amato, 2001

########## Type host.

*Parastacus brasiliensis* (von Martens, 1869) (Decapoda: Parastacidae).

########## Infection site.

Branchial chamber.

########## Type locality.

Brazil, Rio Grande do Sul State, Taquara (29°37'S, 50°47'W).

########## Holotype.

♀ CHIOC 34334 a.

########## Paratypes.

CHIOC 34334 b (allotype ♂), 34335 a–b, e (♀♀), c–d, f (♂♂).

########## Remarks.

Other paratypes deposited in the USNM collection.

########## Reference.

Amato (2001).

##### Class Hirudinea Linnaeus, 1758

###### Order Rhynchobdellida (Blanchard, 1894)

####### Ozobranchidae (Pinto, 1921)

######## *Unoculubranchiobdella* Peralta, Matos & Serra-Freire, 1998

######### * Unoculubranchiobdella expansa

Taxon classificationAnimaliaRhynchobdellidaOzobranchidae

Peralta, Matos & Serra-Freire, 1998

########## Type host.

*Podocnemis expansa* (Schweigger, 1812) (Testudines: Podocnemididae).

########## Infection site.

Carapace, plastron, head, over the eyes, pelvic appendages, cloaca, mouth and nostrils.

########## Type locality.

Brazil, Pará State, Belém, Zoobotanical Park of the Emílio Goeldi Museum.

########## Holotype.

CHIOC 37524 a.

########## Paratypes.

CHIOC 33598, 37524 b–e.

########## Remarks.

The holotype and some paratypes received new numbers because part of the type material was mounted from CHIOC 33598 by the authors after the publication. CHIOC 33598 was indicated as holotype and paratypes in the original description.

########## Reference.

Peralta et al. (1998).

#### Phylum Arthropoda von Siebold, 1848

##### Class Maxillopoda Dahl, 1956

###### Subclass Copepoda Milne Edwards, 1840

####### Order Poecilostomatoida Thorell, 1859

######## Bomolochidae Sumpf, 1871

######### *Hamaticolax* Ho & Lin, 2006

########## Hamaticolax unisagittatus

Taxon classificationAnimaliaPoecilostomatoidaBomolochidae

(Tavares & Luque, 2003)

########### Type host.

*Centropomus undecimalis* (Block, 1792) (Osteichthyes: Centropomidae).

########### Infection site.

Gills.

########### Type locality.

Brazil, Rio de Janeiro State, Angra dos Reis (23°01'S, 44°19'W).

########### Paratypes.

CHIOC 34819 (10♀♀).

########### Remarks.

Species placed in *Acantholochus* Cressey, 1984 by Tavares and Luque (2003) and transferred to *Hamaticolax* by Morales-Serna and Gómez (2010). Holotype and other female paratypes deposited in the MNRJ carcinological collection.

########### References.

Tavares and Luque (2003), Morales-Serna and Gómez (2010).

######## Chondracanthidae Milne Edwards, 1840

######### *Pseudolernentoma* Luque & Alves, 2003

########## * Pseudolernentoma brasiliensis

Taxon classificationAnimaliaPoecilostomatoidaChondracanthidae

Luque & Alves, 2003

########### Type host.

*Genypterus brasiliensis* Regan, 1903 (Osteichthyes: Ophidiidae).

########### Infection site.

Oral cavity.

########### Type locality.

Brazil, coastal zone of the Rio de Janeiro State (21–23°S, 41–45°W).

########### Paratypes.

CHIOC 34892 (5♀♀), 34893 (3♂♂).

########### Remarks.

Female holotype, male allotype, and other male and female paratypes deposited in the MNRJ carcinological collection.

########### Reference.

Luque and Alves (2003).

######## Ergasilidae von Nordmann, 1832

######### *Brasergasilus* Thatcher & Boeger, 1983

########## Brasergasilus bifurcatus

Taxon classificationAnimaliaPoecilostomatoidaErgasilidae

Santos, Thatcher & Brasil-Sato, 2007

########### Type host.

*Pygocentrus piraya* (Cuvier, 1819)

########### Infection site.

Gill filaments and nasal fossae.

########### Type locality.

Brazil, Minas Gerais State, Upper São Francisco River, Três Marias Reservoir (18°12'59"S, 45°17'34"W).

########### Holotype.

♀ CHIOC 36841.

########### Paratypes.

CHIOC 35502 (♀), 35503 (♀), 35504 (♀), 36842 (♀), 36843 (♀), 36844 (♀).

########### Remarks.

CHIOC 35504, 36843, and 36844 collected from the serrasalmid fish *Serrasalmus brandtii* Lütken, 1875.

########### Reference.

Santos et al. (2007).

######### *Ergasilus* von Nordmann, 1832

########## Ergasilus thatcheri

Taxon classificationAnimaliaPoecilostomatoidaErgasilidae

Engers, Boeger & Brandão, 2000

########### Type host.

*Rhamdia quelen* (Quoy & Gaimard, 1824) (Osteichthyes: Heptapteridae).

########### Infection site.

Gills filaments.

########### Type locality.

Brazil, Rio Grande do Sul State, Barragem do Capané, Cachoeira do Sul.

########### Holotype.

♀ CHIOC 34008 a.

########### Paratypes.

CHIOC 33840 a–c, 34008 b–f (♀♀).

########### Remarks.

Other paratypes deposited in the collections of HWML and USNM.

########### Reference.

Engers et al. (2000).

########## Ergasilus youngi

Taxon classificationAnimaliaPoecilostomatoidaErgasilidae

Tavares & Luque, 2005

########### Type host.

*Aspistor luniscutis* (Valenciennes, 1840) (Osteichthyes: Ariidae).

########### Infection site.

Gills.

########### Type locality.

Brazil, Rio de Janeiro State, Angra dos Reis (23°01'S, 44°19'W).

########### Holotype.

♀ CHIOC 35393.

########### Paratypes.

CHIOC 35392 (5♀♀), 35394 (5♀♀).

########### Reference.

Tavares and Luque (2005).
